# *N*-{2-[4-(2-Meth­oxy­phen­yl)piperazin-1-yl]eth­yl}pyridin-2-amine monohydrate

**DOI:** 10.1107/S1600536810022816

**Published:** 2010-06-23

**Authors:** Quan-Fu Jiang, Chun-Xiong Lu

**Affiliations:** a Key Laboratory of Nuclear Medicine, Ministry of Health, Jiangsu Key Laboratory of Molecular Nuclear Medicine, Jiangsu Institute of Nuclear Medicine, Wuxi 214063, People’s Republic of China

## Abstract

In the title compound, C_18_H_24_N_4_O·H_2_O, the piperizine ring adopts a chair conformation and the dihedral angle between the phenyl and pyridine rings is 39.9 (3)°. The comformations of the attachment of the anisole and *N*-ethyl­pyridin-2-amine groups to the piperazine ring are +anti­periplanar. An intra­molecular C—H⋯O inter­action occurs. In the crystal, the water mol­ecule links the mol­ecules into chains through O—H⋯N hydrogen bonds. Weak N—H⋯O, C—H⋯N and C—H⋯O inter­actions further stabilize the crystal structure.

## Related literature

For the use of the title compound in the synthesis of receptor imaging agents, see: Lebars *et al.* (1998 ▶); Zhuang *et al.* (1994 ▶).
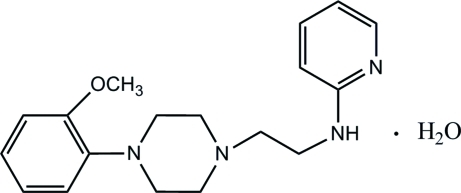


## Experimental

### 

#### Crystal data

C_18_H_24_N_4_O·H_2_O
*M* *_r_* = 330.43
Orthorhombic, 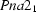

*a* = 13.451 (3) Å
*b* = 19.847 (4) Å
*c* = 6.8596 (15) Å
*V* = 1831.2 (7) Å^3^
*Z* = 4
Mo *K*α radiation
μ = 0.08 mm^−1^
*T* = 153 K
0.40 × 0.23 × 0.09 mm

#### Data collection

Rigaku R-AXIS Spider diffractometer
14086 measured reflections
2261 independent reflections
1985 reflections with *I* > 2σ(*I*)
*R*_int_ = 0.049

#### Refinement

*R*[*F* ^2^ > 2σ(*F* ^2^)] = 0.042
*wR*(*F* ^2^) = 0.091
*S* = 1.00
2261 reflections
230 parameters
1 restraint
H atoms treated by a mixture of independent and constrained refinement
Δρ_max_ = 0.18 e Å^−3^
Δρ_min_ = −0.15 e Å^−3^

### 

Data collection: *RAPID-AUTO* (Rigaku, 2004 ▶); cell refinement: *RAPID-AUTO*; data reduction: *RAPID-AUTO*; program(s) used to solve structure: *SHELXS97* (Sheldrick, 2008 ▶); program(s) used to refine structure: *SHELXL97* (Sheldrick, 2008 ▶); molecular graphics: *SHELXTL* (Sheldrick, 2008 ▶); software used to prepare material for publication: *SHELXTL*.

## Supplementary Material

Crystal structure: contains datablocks I, global. DOI: 10.1107/S1600536810022816/pv2288sup1.cif

Structure factors: contains datablocks I. DOI: 10.1107/S1600536810022816/pv2288Isup2.hkl

Additional supplementary materials:  crystallographic information; 3D view; checkCIF report

## Figures and Tables

**Table 1:** Hydrogen-bond geometry (Å, °)

*D*—H⋯*A*	*D*—H	H⋯*A*	*D*⋯*A*	*D*—H⋯*A*
O2—H0*A*⋯N1^i^	0.87 (3)	2.01 (3)	2.877 (3)	179 (4)
O2—H0*B*⋯N3	0.83 (3)	2.01 (3)	2.831 (2)	174 (3)
N2—H2*N*⋯O2^i^	0.83 (3)	2.05 (3)	2.864 (3)	168 (3)
C3—H3⋯N4^ii^	0.95	2.56	3.471 (3)	161
C10—H10*A*⋯O1	0.99	2.36	2.957 (3)	118
C15—H15⋯O2^iii^	0.95	2.58	3.379 (3)	142

Symmetry codes: (i) 
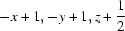
; (ii) 
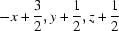
; (iii) 
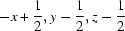
.

**Fig. 1.**
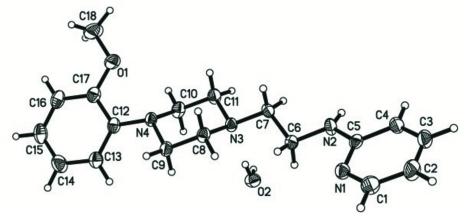
A view of the title compound with the atomic numbering scheme. Displacement ellipsoids were drawn at the 50% probability level.

## supplementary crystallographic information

### Comment

*N*-(2-(4-(2-Methoxyphenyl)piperazin-1-yl)ethyl)pyridin-2-amine, (I), is an important intermediate product in the synthesis of ^131^I-MPPI (Zhuang *et al.*, 1994) and ^18^F-MPPF (Lebars *et al.*, 1998), serotonin(5-HT_1A_) receptor imaging agents (^131^I-MPPI = 4-(2'-methoxypheny)-1-[2'-(*N*-2''-pyridinyl)- p-^131^I-iodobenzamido]ethyl-piperazine and ^18^F-MPPF = 4-(2'-methoxyphenyl)-1-[2'-(*N*-2''-pyridinyl) -p-^18^F-fluorobenzamido]ethylpiperazine). We report here the crystal structure of (I).hydrate (Fig. 1). The molecule of (I) consists of an anisole and an *N*-ethylpyridin-2-amine arms connected to a piperazine ring. The piperazine ring adopts a chair conformation. The dihedral angle between the phenyl and pyridine rings is 39.9 (3)°. The comformation of the attachment of the anisole and *N*-ethylpyridin-2-amine groups to the piperazine ring are best described by the torsion angles of 168.35 (19)° and 179.45 (17)° for C12—N4—C10—C11 and C7—N3—C8—C9, respectively; *i.e.* they adopt +antiperiplanar conformations. The molecules are linked through hydrogen-bonding interactions of types O—H···O, N—H···O and C—H···O (Table 1).

### Experimental

The title compound was synthesized according to the method reported in the literature (Zhuang *et al.*, 1994) and crystallized from a mixed solvent composed of acetone and water (1:1); colorless block-shaped crystals were obtained after several days.

### Refinement

An absolute structure could not be determined dfue to lack of sufficient dispersion effects. Therefore, Friedel pairs (1894) were merged. Positional parameters of all the H atoms bonded to C atoms were calculated geometrically and were allowed to ride on the C atoms to which they were bonded, with C—H distances of 0.95Å (CH), 0.98Å (CH_3_) or 0.99Å (CH_2_), and with *U*_iso_(H) = 1.2U_eq_ of the parent atoms. The H-atoms bonded to N and O atoms were taken from a difference map and were allowed to refine freely.

### Crystal data

**Table:**

C_18_H_24_N_4_O·H_2_O	*F*(000) = 712
*M**_r_* = 330.43	*D*_x_ = 1.199 Mg m^−^^3^
Orthorhombic, *P**n**a*2_1_	Mo *K*α radiation, λ = 0.71073 Å
Hall symbol: P 2c -2n	Cell parameters from 4880 reflections
*a* = 13.451 (3) Å	θ = 3.0–27.5°
*b* = 19.847 (4) Å	µ = 0.08 mm^−^^1^
*c* = 6.8596 (15) Å	*T* = 153 K
*V* = 1831.2 (7) Å^3^	Prism, colorless
*Z* = 4	0.40 × 0.23 × 0.09 mm

### Data collection

**Table:**

Rigaku R-AXIS Spider diffractometer	1985 reflections with *I* > 2σ(*I*)
Radiation source: Rotating Anode	*R*_int_ = 0.049
graphite	θ_max_ = 27.5°, θ_min_ = 3.0°
ω scans	*h* = −17→17
14086 measured reflections	*k* = −25→22
2261 independent reflections	*l* = −8→8

### Refinement

**Table:**

Refinement on *F*^2^	Primary atom site location: structure-invariant direct methods
Least-squares matrix: full	Secondary atom site location: difference Fourier map
*R*[*F*^2^ > 2σ(*F*^2^)] = 0.042	Hydrogen site location: inferred from neighbouring sites
*wR*(*F*^2^) = 0.091	H atoms treated by a mixture of independent and constrained refinement
*S* = 1.00	*w* = 1/[σ^2^(*F*_o_^2^) + (0.0505*P*)^2^ + 0.160*P*] where *P* = (*F*_o_^2^ + 2*F*_c_^2^)/3
2261 reflections	(Δ/σ)_max_ < 0.001
230 parameters	Δρ_max_ = 0.18 e Å^−^^3^
1 restraint	Δρ_min_ = −0.15 e Å^−^^3^

### Special details

**Table:**

Geometry. All e.s.d.'s (except the e.s.d. in the dihedral angle between two l.s. planes) are estimated using the full covariance matrix. The cell e.s.d.'s are taken into account individually in the estimation of e.s.d.'s in distances, angles and torsion angles; correlations between e.s.d.'s in cell parameters are only used when they are defined by crystal symmetry. An approximate (isotropic) treatment of cell e.s.d.'s is used for estimating e.s.d.'s involving l.s. planes.
Refinement. Refinement of *F*^2^ against ALL reflections. The weighted *R*-factor *wR* and goodness of fit *S* are based on *F*^2^, conventional *R*-factors *R* are based on *F*, with *F* set to zero for negative *F*^2^. The threshold expression of *F*^2^ > σ(*F*^2^) is used only for calculating *R*-factors(gt) *etc*. and is not relevant to the choice of reflections for refinement. *R*-factors based on *F*^2^ are statistically about twice as large as those based on *F*, and *R*- factors based on ALL data will be even larger.

### Fractional atomic coordinates and isotropic or equivalent isotropic displacement parameters (Å^2^)

**Table:**

	*x*	*y*	*z*	*U*_iso_*/*U*_eq_	
O1	0.43965 (12)	0.13354 (8)	0.6019 (3)	0.0352 (4)	
O2	0.35365 (13)	0.46544 (8)	0.6564 (3)	0.0296 (4)	
N1	0.75758 (14)	0.58179 (9)	0.4876 (3)	0.0254 (4)	
N2	0.69260 (15)	0.52222 (10)	0.7505 (3)	0.0258 (4)	
N3	0.52175 (13)	0.38007 (8)	0.6319 (3)	0.0218 (4)	
N4	0.44650 (13)	0.25939 (8)	0.4558 (3)	0.0222 (4)	
C1	0.82362 (19)	0.62843 (12)	0.4242 (4)	0.0341 (6)	
H1	0.8274	0.6364	0.2879	0.041*	
C2	0.88518 (19)	0.66487 (13)	0.5425 (4)	0.0357 (6)	
H2	0.9308	0.6966	0.4900	0.043*	
C3	0.87886 (17)	0.65392 (12)	0.7421 (4)	0.0316 (6)	
H3	0.9195	0.6789	0.8293	0.038*	
C4	0.81411 (17)	0.60715 (11)	0.8117 (3)	0.0267 (5)	
H4	0.8095	0.5990	0.9479	0.032*	
C5	0.75372 (15)	0.57077 (10)	0.6798 (3)	0.0212 (5)	
C6	0.62161 (16)	0.48524 (10)	0.6333 (3)	0.0257 (5)	
H6A	0.6490	0.4776	0.5012	0.031*	
H6B	0.5593	0.5115	0.6204	0.031*	
C7	0.60037 (17)	0.41825 (11)	0.7313 (3)	0.0251 (5)	
H7A	0.6620	0.3910	0.7333	0.030*	
H7B	0.5802	0.4265	0.8681	0.030*	
C8	0.55437 (16)	0.35756 (11)	0.4378 (3)	0.0228 (5)	
H8A	0.6145	0.3291	0.4509	0.027*	
H8B	0.5718	0.3972	0.3572	0.027*	
C9	0.47335 (16)	0.31789 (10)	0.3383 (3)	0.0243 (5)	
H9A	0.4142	0.3469	0.3195	0.029*	
H9B	0.4966	0.3029	0.2084	0.029*	
C10	0.41288 (17)	0.28054 (11)	0.6490 (3)	0.0283 (5)	
H10A	0.3967	0.2404	0.7288	0.034*	
H10B	0.3520	0.3083	0.6367	0.034*	
C11	0.49388 (19)	0.32103 (11)	0.7477 (3)	0.0286 (5)	
H11A	0.4704	0.3359	0.8776	0.034*	
H11B	0.5530	0.2921	0.7671	0.034*	
C12	0.38607 (15)	0.21023 (10)	0.3614 (3)	0.0243 (5)	
C13	0.33403 (17)	0.22331 (11)	0.1915 (4)	0.0300 (5)	
H13	0.3335	0.2678	0.1403	0.036*	
C14	0.28283 (19)	0.17297 (13)	0.0947 (4)	0.0394 (6)	
H14	0.2476	0.1831	−0.0218	0.047*	
C15	0.28287 (19)	0.10831 (13)	0.1670 (4)	0.0388 (6)	
H15	0.2484	0.0736	0.0997	0.047*	
C16	0.33326 (18)	0.09392 (12)	0.3380 (4)	0.0338 (6)	
H16	0.3322	0.0494	0.3889	0.041*	
C17	0.38516 (16)	0.14392 (11)	0.4357 (4)	0.0274 (5)	
C18	0.4404 (2)	0.06745 (13)	0.6815 (5)	0.0505 (8)	
H18A	0.3720	0.0530	0.7072	0.061*	
H18B	0.4782	0.0674	0.8036	0.061*	
H18C	0.4715	0.0364	0.5886	0.061*	
H0A	0.320 (2)	0.4515 (15)	0.757 (5)	0.051 (9)*	
H2N	0.686 (2)	0.5217 (13)	0.871 (4)	0.033 (7)*	
H0B	0.400 (2)	0.4384 (17)	0.647 (6)	0.063 (11)*	

### Atomic displacement parameters (Å^2^)

**Table:**

	*U*^11^	*U*^22^	*U*^33^	*U*^12^	*U*^13^	*U*^23^
O1	0.0412 (9)	0.0224 (8)	0.0420 (10)	−0.0083 (7)	−0.0081 (9)	0.0084 (8)
O2	0.0339 (9)	0.0310 (9)	0.0239 (9)	0.0051 (8)	0.0037 (8)	0.0022 (7)
N1	0.0265 (10)	0.0241 (10)	0.0257 (10)	−0.0036 (8)	−0.0005 (8)	−0.0006 (8)
N2	0.0314 (10)	0.0257 (9)	0.0203 (10)	−0.0092 (8)	−0.0007 (9)	−0.0035 (8)
N3	0.0277 (9)	0.0194 (8)	0.0183 (9)	−0.0054 (7)	0.0004 (8)	0.0000 (7)
N4	0.0256 (9)	0.0182 (8)	0.0228 (9)	−0.0045 (7)	−0.0002 (8)	−0.0013 (8)
C1	0.0380 (14)	0.0343 (13)	0.0300 (13)	−0.0063 (11)	0.0025 (11)	0.0049 (11)
C2	0.0341 (13)	0.0321 (13)	0.0410 (15)	−0.0149 (11)	0.0015 (12)	0.0029 (11)
C3	0.0253 (11)	0.0277 (12)	0.0418 (14)	−0.0039 (10)	−0.0071 (11)	−0.0046 (11)
C4	0.0261 (11)	0.0277 (11)	0.0263 (12)	−0.0004 (10)	−0.0034 (10)	−0.0037 (10)
C5	0.0206 (10)	0.0175 (10)	0.0256 (11)	0.0019 (8)	0.0008 (9)	−0.0023 (9)
C6	0.0300 (11)	0.0206 (10)	0.0264 (12)	−0.0066 (9)	−0.0063 (10)	0.0021 (9)
C7	0.0306 (11)	0.0240 (11)	0.0208 (11)	−0.0045 (9)	−0.0066 (10)	−0.0002 (9)
C8	0.0256 (10)	0.0197 (10)	0.0231 (11)	−0.0043 (9)	0.0033 (10)	0.0005 (9)
C9	0.0289 (11)	0.0198 (10)	0.0242 (11)	−0.0032 (9)	−0.0005 (10)	−0.0014 (9)
C10	0.0350 (12)	0.0241 (11)	0.0258 (12)	−0.0061 (9)	0.0079 (11)	−0.0004 (10)
C11	0.0409 (13)	0.0247 (11)	0.0204 (11)	−0.0102 (10)	−0.0004 (11)	0.0019 (10)
C12	0.0203 (10)	0.0233 (10)	0.0292 (12)	−0.0034 (9)	0.0019 (10)	−0.0044 (10)
C13	0.0307 (12)	0.0273 (12)	0.0320 (13)	−0.0040 (10)	−0.0009 (11)	−0.0030 (10)
C14	0.0384 (13)	0.0416 (14)	0.0381 (14)	−0.0114 (11)	−0.0092 (13)	−0.0024 (13)
C15	0.0379 (13)	0.0358 (13)	0.0427 (14)	−0.0144 (11)	−0.0008 (13)	−0.0110 (12)
C16	0.0330 (13)	0.0237 (11)	0.0448 (15)	−0.0093 (10)	0.0057 (12)	−0.0036 (11)
C17	0.0243 (11)	0.0245 (11)	0.0335 (13)	−0.0024 (9)	0.0026 (10)	−0.0029 (10)
C18	0.0586 (18)	0.0296 (13)	0.063 (2)	−0.0095 (12)	−0.0168 (17)	0.0175 (14)

### Geometric parameters (Å, °)

**Table:**

O1—C17	1.371 (3)	C7—H7A	0.9900
O1—C18	1.421 (3)	C7—H7B	0.9900
O2—H0A	0.88 (3)	C8—C9	1.508 (3)
O2—H0B	0.83 (3)	C8—H8A	0.9900
N1—C5	1.338 (3)	C8—H8B	0.9900
N1—C1	1.354 (3)	C9—H9A	0.9900
N2—C5	1.356 (3)	C9—H9B	0.9900
N2—C6	1.448 (3)	C10—C11	1.514 (3)
N2—H2N	0.83 (3)	C10—H10A	0.9900
N3—C11	1.464 (3)	C10—H10B	0.9900
N3—C7	1.469 (3)	C11—H11A	0.9900
N3—C8	1.472 (3)	C11—H11B	0.9900
N4—C12	1.426 (3)	C12—C13	1.384 (3)
N4—C9	1.459 (3)	C12—C17	1.411 (3)
N4—C10	1.462 (3)	C13—C14	1.383 (3)
C1—C2	1.366 (4)	C13—H13	0.9500
C1—H1	0.9500	C14—C15	1.376 (4)
C2—C3	1.389 (4)	C14—H14	0.9500
C2—H2	0.9500	C15—C16	1.385 (4)
C3—C4	1.360 (3)	C15—H15	0.9500
C3—H3	0.9500	C16—C17	1.386 (3)
C4—C5	1.414 (3)	C16—H16	0.9500
C4—H4	0.9500	C18—H18A	0.9800
C6—C7	1.517 (3)	C18—H18B	0.9800
C6—H6A	0.9900	C18—H18C	0.9800
C6—H6B	0.9900		
			
C17—O1—C18	117.5 (2)	H8A—C8—H8B	108.1
H0A—O2—H0B	105 (3)	N4—C9—C8	110.15 (18)
C5—N1—C1	117.0 (2)	N4—C9—H9A	109.6
C5—N2—C6	124.1 (2)	C8—C9—H9A	109.6
C5—N2—H2N	115.1 (19)	N4—C9—H9B	109.6
C6—N2—H2N	118.7 (19)	C8—C9—H9B	109.6
C11—N3—C7	110.20 (17)	H9A—C9—H9B	108.1
C11—N3—C8	108.93 (16)	N4—C10—C11	109.59 (18)
C7—N3—C8	111.22 (17)	N4—C10—H10A	109.8
C12—N4—C9	115.76 (18)	C11—C10—H10A	109.8
C12—N4—C10	115.61 (17)	N4—C10—H10B	109.8
C9—N4—C10	110.42 (16)	C11—C10—H10B	109.8
N1—C1—C2	124.7 (2)	H10A—C10—H10B	108.2
N1—C1—H1	117.7	N3—C11—C10	111.48 (19)
C2—C1—H1	117.7	N3—C11—H11A	109.3
C1—C2—C3	117.7 (2)	C10—C11—H11A	109.3
C1—C2—H2	121.1	N3—C11—H11B	109.3
C3—C2—H2	121.1	C10—C11—H11B	109.3
C4—C3—C2	119.5 (2)	H11A—C11—H11B	108.0
C4—C3—H3	120.3	C13—C12—C17	118.3 (2)
C2—C3—H3	120.3	C13—C12—N4	122.9 (2)
C3—C4—C5	119.5 (2)	C17—C12—N4	118.6 (2)
C3—C4—H4	120.3	C14—C13—C12	121.4 (2)
C5—C4—H4	120.3	C14—C13—H13	119.3
N1—C5—N2	119.5 (2)	C12—C13—H13	119.3
N1—C5—C4	121.7 (2)	C15—C14—C13	120.0 (2)
N2—C5—C4	118.8 (2)	C15—C14—H14	120.0
N2—C6—C7	108.79 (18)	C13—C14—H14	120.0
N2—C6—H6A	109.9	C14—C15—C16	119.9 (2)
C7—C6—H6A	109.9	C14—C15—H15	120.1
N2—C6—H6B	109.9	C16—C15—H15	120.1
C7—C6—H6B	109.9	C15—C16—C17	120.6 (2)
H6A—C6—H6B	108.3	C15—C16—H16	119.7
N3—C7—C6	112.45 (17)	C17—C16—H16	119.7
N3—C7—H7A	109.1	O1—C17—C16	124.3 (2)
C6—C7—H7A	109.1	O1—C17—C12	115.84 (19)
N3—C7—H7B	109.1	C16—C17—C12	119.8 (2)
C6—C7—H7B	109.1	O1—C18—H18A	109.5
H7A—C7—H7B	107.8	O1—C18—H18B	109.5
N3—C8—C9	110.64 (18)	H18A—C18—H18B	109.5
N3—C8—H8A	109.5	O1—C18—H18C	109.5
C9—C8—H8A	109.5	H18A—C18—H18C	109.5
N3—C8—H8B	109.5	H18B—C18—H18C	109.5
C9—C8—H8B	109.5		
			
C5—N1—C1—C2	−0.5 (4)	C7—N3—C11—C10	−179.99 (19)
N1—C1—C2—C3	−0.8 (4)	C8—N3—C11—C10	−57.7 (2)
C1—C2—C3—C4	1.3 (4)	N4—C10—C11—N3	58.0 (2)
C2—C3—C4—C5	−0.5 (4)	C9—N4—C12—C13	−16.5 (3)
C1—N1—C5—N2	−176.7 (2)	C10—N4—C12—C13	114.8 (2)
C1—N1—C5—C4	1.4 (3)	C9—N4—C12—C17	158.5 (2)
C6—N2—C5—N1	−7.0 (3)	C10—N4—C12—C17	−70.1 (3)
C6—N2—C5—C4	174.9 (2)	C17—C12—C13—C14	−0.6 (3)
C3—C4—C5—N1	−0.9 (3)	N4—C12—C13—C14	174.5 (2)
C3—C4—C5—N2	177.2 (2)	C12—C13—C14—C15	0.1 (4)
C5—N2—C6—C7	155.8 (2)	C13—C14—C15—C16	0.8 (4)
C11—N3—C7—C6	−171.59 (19)	C14—C15—C16—C17	−1.1 (4)
C8—N3—C7—C6	67.5 (2)	C18—O1—C17—C16	−1.8 (3)
N2—C6—C7—N3	174.53 (18)	C18—O1—C17—C12	179.8 (2)
C11—N3—C8—C9	57.8 (2)	C15—C16—C17—O1	−177.7 (2)
C7—N3—C8—C9	179.45 (17)	C15—C16—C17—C12	0.6 (4)
C12—N4—C9—C8	−167.51 (17)	C13—C12—C17—O1	178.7 (2)
C10—N4—C9—C8	58.7 (2)	N4—C12—C17—O1	3.4 (3)
N3—C8—C9—N4	−58.9 (2)	C13—C12—C17—C16	0.3 (3)
C12—N4—C10—C11	168.35 (19)	N4—C12—C17—C16	−175.0 (2)
C9—N4—C10—C11	−57.8 (2)		

### Hydrogen-bond geometry (Å, °)

**Table:**

*D*—H···*A*	*D*—H	H···*A*	*D*···*A*	*D*—H···*A*
O2—H0A···N1^i^	0.87 (3)	2.01 (3)	2.877 (3)	179 (4)
O2—H0B···N3	0.83 (3)	2.01 (3)	2.831 (2)	174 (3)
N2—H2N···O2^i^	0.83 (3)	2.05 (3)	2.864 (3)	168 (3)
C3—H3···N4^ii^	0.95	2.56	3.471 (3)	161
C10—H10A···O1	0.99	2.36	2.957 (3)	118
C15—H15···O2^iii^	0.95	2.58	3.379 (3)	142

Symmetry codes: (i) −*x*+1, −*y*+1, *z*+1/2; (ii) −*x*+3/2, *y*+1/2, *z*+1/2; (iii) −*x*+1/2, *y*−1/2, *z*−1/2.
